# The NRF2 antagonist ML385 inhibits PI3K‐mTOR signaling and growth of lung squamous cell carcinoma cells

**DOI:** 10.1002/cam4.5311

**Published:** 2022-10-28

**Authors:** Lili Ji, Nadeem Moghal, Xinru Zou, Yixuan Fang, Shuning Hu, Yuhui Wang, Ming Sound Tsao

**Affiliations:** ^1^ Department of Pathology, Key Laboratory of Microenvironment and Translational Cancer Research Medical School of Nantong University Nantong Jiangsu China; ^2^ Princess Margaret Cancer Centre University Health Network Toronto Ontario Canada; ^3^ Department of Medical Biophysics University of Toronto Toronto Ontario Canada

**Keywords:** lung squamous cell carcinoma, mTOR, NRF2, organoid, tumor growth

## Abstract

**Background:**

Lung squamous cell carcinoma (LUSC) currently has limited therapeutic options because of the relatively few validated targets and the lack of clinical drugs for some of these targets. Although NRF2/NFE2L2 pathway activation commonly occurs in LUSC, NRF2 has predominantly been studied in other cancer models. Here, we investigated the function of NRF2 in LUSC, including in organoid models, and we explored the activity of a small molecule NRF2 inhibitor ML385, which has not previously been investigated in LUSC.

**Methods:**

We first explored the role of NRF2 signaling in LUSC cancer cell line and organoid proliferation through NRF2 knockdown or ML385 treatment, both in vivo and in vitro. Next, we performed Western blot and immunofluorescence assays to determine the effect of NRF2 inhibition on PI3K‐mTOR signaling. Finally, we used cell viability and clonogenic assays to explore whether ML385 could sensitize LUSC cancer cells to PI3K inhibitors.

**Results:**

We find that downregulation of NRF2 signaling inhibited proliferation of LUSC cancer cell lines and organoids, both in vivo and in vitro. We also demonstrate that inhibition of NRF2 reduces PI3K‐mTOR signaling, with two potential mechanisms being involved. Although NRF2 promotes AKT phosphorylation, it also acts downstream of AKT to increase RagD protein expression and recruitment of mTOR to lysosomes after amino acid stimulation. We also find that ML385 potentiates LUSC growth inhibition by a pan‐PI3K inhibitor, which correlates with stronger inhibition of PI3K‐mTOR signaling.

**Conclusions:**

Our data provide additional support for NRF2 promoting LUSC growth through PI3K‐mTOR activation and support development of NRF2 inhibitors for the treatment of LUSC.

## Graphical abstract

The NRF2 antagonist ML385 inhibits PI3K‐mTOR signaling through inhibition of RagD protein expression and recruitment of mTOR to lysosomes after amino acid stimulation. Therefore, ML385 inhibits growth of lung squamous cell carcinoma cells. (The parts our study shows have been highlighted in red arrows).

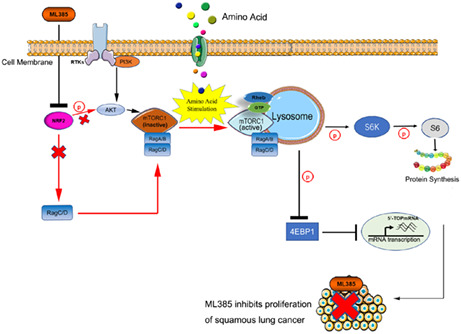

## 1 INTRODUCTION

Lung cancer is the second most commonly diagnosed cancer (11.4% of total cases) and is the leading cause of cancer death (18% of total cancer deaths) worldwide.^1^ Non‐small cell lung cancer (NSCLC) is the most common form of lung cancer, with lung adenocarcinoma (LUAD) and lung squamous cell carcinoma (LUSC) being the predominant forms.^2^ For LUAD, the most common type of NSCLC, drugs that target specific mutated drivers such as EGFR,^3^ ALK,^4^ and KRAS^5^ have been developed and display remarkable therapeutic effects. However, activating mutations in *EGFR*, *ALK*, and *KRAS* are typically not present in the second most common type of NSCLC, LUSC. Presumably, the low frequency or absence of these targetable driver alterations in LUSC accounts for the ineffectiveness of these treatments in LUSC. Therefore, identifying new therapeutic strategies for LUSC remains a high priority.

The redox sensitive bZIP transcription factor, nuclear factor erythroid 2‐related factor 2 (NFE2L2 or NRF2), is a “master regulator” of cell survival through its coordinated induction of cytoprotective antioxidant genes, phase‐II detoxification enzymes, multidrug transporters, and central metabolic pathways.^6^, ^7^ Kelch‐like ECH‐associated protein 1 (KEAP1) negatively regulates NRF2 by directly binding to and promoting its proteasomal degradation. Constitutive activation/stabilization of NRF2 has been reported in various human cancers, including NSCLC.^8^, ^9^ Activating mutations in *NRF2*, which impair its protein binding to KEAP1, have been identified in LUSC.^10^, ^11^ Also, comprehensive genomics data from The Cancer Genome Atlas (TCGA) and other clinical data sets have documented extensive alterations in the NRF2 pathway in LUSC patients, with a cumulative frequency of ~34%.^10^, ^12^, ^13^ In addition, NRF2 is more commonly expressed in LUSC (38%) than in LUAD (18%) and is associated with a poor outcome.^14^ This is likely due to NRF2 being a bona‐fide driver for the LUSC since its knockdown in several *NRF2*‐mutated LUSC cell lines inhibits proliferation in vitro.^7^ Furthermore, *KEAP1/NRF2* and *TP53* mutations combine to promote LUSC development and radiation resistance.^15^, ^16^ Cancers with high NRF2 levels are associated with poor prognosis not only because of chemo‐ and radio‐resistance^15^, ^17^ but also because of aggressive proliferation.^7^, ^18^, ^19^, ^20^

In lung cancer, the oncogenic activity of NRF2 may also arise through its ability to upregulate PI3K/mTOR pathway activation.^7^, ^18^, ^19^, ^21^, ^22^, ^23^, ^24^ Co‐occurring NRF2‐PI3K pathway alterations are frequent in lung tumors (both squamous cell and adenocarcinoma).^25^ Also, downstream of PI3K, the mTOR Complex 1 (mTORC1) branch of the mammalian target of rapamycin (mTOR) pathway is a major driver of cell growth and is deregulated in NSCLC.^26^, ^27^, ^28^ These findings support NRF2‐PI3K cross‐talk being functionally important for lung cancer pathogenesis. NRF2 appears to promote PI3K‐mTOR pathway activity through several mechanisms. NRF2 regulates mTOR and RagD transcription.^19^, ^24^ Rag proteins comprise a family of four related small guanosine triphosphatases (GTPases) (RagA, RagB, RagC, RagD) that interact with mTORC1 in an amino acid sensitive manner and are necessary for the activation of mTORC1 by amino acids at lysosomes.^29^, ^30^ *RagD* mRNA levels, in particular, were shown to be upregulated by NRF2 in both LUSC and other cancer cell line models where NRF2 also promoted mTORC1 activity.^19^ This mechanism was clinically validated in LUSC patient samples that showed a significant positive correlation between *NRF2* mutation and *RagD* mRNA expression.^21^ The ability of NRF2 to promote PI3K‐mTOR pathway activity seems to be conserved across cell types, with cross‐talk being observed in multiple types of cancer cell lines, as well as normal cells such as melanocytes and hepatocytes.^7^, ^18^, ^19^, ^21^, ^22^, ^23^, ^24^ Overall, it is thought that NRF2 activation both increases PI3K‐mTOR pathway activity and dependency,^16^, ^18^, ^19^ which may also promote more aggressive proliferation through metabolic reprogramming.^7^

Despite the wealth of information implicating NRF2 in carcinogenesis, few studies have been conducted in LUSC cell line models, which have generally been more difficult to establish than for LUAD.^31^ Over the past few years, organoid cultures derived from primary patient tumors and patient‐derived xenografts (PDXs) have emerged as potential solutions for studies in need of more or better cancer models.^32^ These cancer organoids have been utilized for numerous applications,^33^, ^34^ and present major advances for NSCLC research.^35^, ^36^, ^37^ Another prominent advance for the study of NRF2 in NSCLC includes identification of a small molecule chemical inhibitor, ML385, which inhibits NRF2 DNA binding, as well as expression of itself and downstream target genes.^38^ The goals of the present study were to use additional LUSC models, including organoids our group established,^37^ to characterize NRF2 dependency and its potential activation of the PI3K‐mTOR pathway, as well as sensitivity to small molecule NRF2 chemical inhibitors like ML385. Our findings support NRF2 being a targetable vulnerability in LUSC, with stimulation of PI3K‐mTOR signaling being a key feature of its activation.

## 2 MATERIALS AND METHODS

### 2.1 Cell culture and reagents

The two LUSC cell lines with *NRF2* mutations (EBC1 and LK2) were purchased from the Japanese Collection of Research Bioresources Cell Bank (JCRB0820 and JCRB0829 respectively). The establishment and characterization of the MGH7 cell line has been previously described.^31^, ^39^ Cell lines were cultured in RPMI‐1640 medium supplemented with 10% fetal bovine serum (FBS). Establishment of the LUSC organoid models XDO274 and XDO377 from patient‐derived xenograft (PDX) tumor tissue has also been previously reported.^37^, ^40^, ^41^, ^42^ All cell lines and organoids were authenticated by short tandem repeat profiling and found to be free of mycoplasma contamination by PCR testing. Compounds including ML385 and BKM120 were purchased from the UHN Shanghai Research & Development Co and were dissolved in DMSO. Cisplatin was purchased from Sigma‐Aldrich and was dissolved in DMF.

### 2.2 Organoid immunohistochemical staining

Organoids were fixed in 10% buffered formalin for 24–48 h and then embedded in Histogel (Thermo Fisher Scientific) prior to processing for paraffin embedding. Paraffin blocks of tumor tissues and organoids were cut at 4 μm thickness, and slices were mounted on charged slides and air dried overnight at 60°C. Sections were stained with hematoxylin eosin (H&E) and by immunohistochemistry (IHC) with various antibodies using the BenchMark XT autostainer (Ventana Medical Systems). Primary antibodies used for IHC analysis included those specific to NRF2 (Abcam Cat# ab137550; RRID: AB_2687540; 1:100; Waltham, MA, USA), NQO1 (Santa Cruz Biotechnology Cat# sc‐271,116; RRID: AB_10611356; 1:100; Dallas, TX, USA), phospho‐S6 (CST Cat#4858; 1:500), TP63 (Dako Cat# M7317; 1:600), and TTF‐1 (Dako Cat# M3575; RICD: AB_2877699; 1:400). Slides were scanned and imaged using the Aperio Scanscope XT (Leica).

### 2.3 Western blotting

Organoid tumor colonies growing in Matrigel were dissociated with TrypLE Express (Gibco), while cultured LUSC cells growing in plastic dishes were dissociated with trypsin (Gibco). The organoid or cell line pellets were lysed in RIPA buffer (Sigma) containing phenylmethylsulfonylfluoride, sodium vanadate, and a protease inhibitor cocktail (Roche). Protein was quantified using the Bradford assay (Bio‐Rad), then denatured in sample buffer (Bio‐Rad), and loaded for SDS‐PAGE. Gel‐separated proteins were transferred onto nitrocellulose membranes (Bio‐Rad), blocked in 5% skim milk for 1 hour, and then incubated overnight with appropriate primary antibodies. After washing with Tris‐Buffered Saline Tween‐20 (TBST), the membrane was probed with secondary anti‐rabbit or mouse IgG, then with HRP‐lined antibodies for 1 h prior to imaging. An ECL reagent (GE Healthcare) was used to detect proteins of interest. The primary antibodies used in this study were: NRF2 (#137550, 1:1000 from Abcam), NQO1 (sc‐271,116, 1:1000) and GAPDH (sc‐257,778, 1:1000) (both from Santa Cruz), AKT (#9272, 1:1000), pAKT (Ser 473, #4060, 1:1000), S6 (#2217, 1:5000), pS6 (Ser 235/236, #4858, 1:2000), 4EBP1 (#9644, 1:2000), p4EBP1 (Thr 37/46, #2855, 1:2000), and Rag D (#4470, 1:1000) (all from CST, Danvers), ß‐Actin (#A1978, 1:10000, from Sigma).

### 2.4 NRF2 knockdown in MGH7 cells

*NRF2* shRNA lentiviral constructs in the pLKO.1 and pLKO TRC005 backbones were purchased from Sigma‐Aldrich, St. Louis. The NRF2 targeting sequences included CCGGGCTCCTACTGTGATGTGAAATCTCGAGATTTCACATCACAGTAGGAGCTTTTTG for shRNA1 (284998), CCGGAGTTTGGGAGGAGCTATTATCCTCGAGGATAATAGCTCCTCCCAAACTTTTTTG for shRNA2 (273499), CCGGCCGGCATTTCACTAAACACAACTCGAGTTGTGTTTAGTGAAATGCCGGTTTTT for shRNA3 (007558), CCGGGCACCTTATATCTCGAAGTTTCTCGAGAAACTTCGAGATATAAGGTGCTTTTT for shRNA4 (007556), CCGGGCTCCTACTGTGATGTGAAATCTCGAGATTTCACATCACAGTAGGAGCTTTTT for shRNA5 (007555). The non‐targeting luciferase and lacZ shRNA was used as negative control, with CCGGCAAATCACAGAATCGTCG TATCTCGAGATACGACGATTCTGTGATTTGTTTT TGAATTC for shLuc (TRCN0000072246) and CCGGCCG TCATAGCGATAACGAGTTCTCGAGAACTCGTTATC GCTATGACGGTTTTTG for shLacZ (TRCN0000072235). The NRF2‐shRNA plasmids and negative control plasmid, along with standard helper packaging plasmids,^43^ were transfected into 293 T cells to make lentiviruses (KCB Catalog #KCB 200744YJ; RRID: CVCL_0063) using FuGENE6 (Promega), following the manufacturer's instructions. The viral supernatants were applied to MGH7 cells and after 48 h, cells were subjected to puromycin selection (Sigma‐Aldrich, 3 μg/ml) for 72 h.

### 2.5 Amino acid starvation/stimulation

LK2 cells were rinsed with PBS and incubated in amino acid‐free RPMI supplemented with 10% dialyzed FBS for 50 min, and then left untreated or stimulated with RPMI 1640 media containing a mixture of essential and nonessential amino acids for 15 min. Cells were processed for immunofluorescence assays as described below.

### 2.6 Immunofluorescence assays

For detection of the localization of mTOR, RagD, and LAMP1, LK2 cells were plated on glass coverslips in 24‐well tissue culture plates and treated with DMSO or ML385 (5 μM) for 48 h. Cells were then starved for amino acids and stimulated with amino acids as described above. Cells were then rinsed three times with PBS and fixed for 20 min with 4% paraformaldehyde in PBS at room temperature and rinsed three more times with PBS. Next, the coverslips were blocked for 45 min in blocking buffer (0.3% Triton‐X100 plus 5% donkey serum in PBS) and incubated with primary antibodies for mTOR (R&D systems, Minneapolis, MN, USA; MAB1537, 1:200), RagD (Cell Signaling Technology; #4470, 1:500), or LAMP1 (Santa Cruz Biotechnology; sc‐20,011, 1:100) in blocking buffer (1% BSA plus 1% donkey serum in PBS) overnight at 4°C. The next day, the coverslips were rinsed with PBS three times and incubated with secondary antibodies (Thermo Fisher Scientific; Alexa Fluor® 568 Donkey Anti‐Rabbit IgG Antibody A‐10042, Alexa Fluor® 488 Donkey Anti‐Rabbit IgG Antibody A‐21206, and Alexa Fluor® 488 Donkey Anti‐Mouse IgG [H + L] Antibody A‐21202) diluted in dilution buffer (1% BSA plus 1% donkey serum in PBS) for 1 h at room temperature in the dark. The coverslips were mounted on glass slides and imaged with a 160‐fold magnification objective using a Leica SP8 confocal microscope. Co‐localization analysis was performed by Fiji software (RRIC: SCR_002285). The scatter plots based on the pixel intensity values of the dual‐channel images were constructed with the coloc 2 plugin in Fiji.^44^, ^45^ The degree of colocalization was then quantified by Pearson's correlation coefficients (PCC), which were calculated using coloc 2 in Fiji^46^ based on the pixel intensity values (including red and green values) collected from the dual‐channel images. The assay was performed three times per condition, and 10 cells were collected from the 3 replicates. Statistical analysis was performed based on the PCC values collected from the 10 cells. All immunofluorescence experiments have been repeated 3 times with representative results shown.

### 2.7 Soft agar colony formation assay

A soft agar colony formation assay was used to assess anchorage independent growth of cancer cells.^47^ Media were prepared as two separate 2× concentrated solutions with noble agar (Sigma‐Aldrich) (2% for the base layer and 0.8% for the cell growth layer), which were then diluted with 2× RPMI prior to being added to dishes. MGH7 cells (1 × 10^4^ cells per 60 × 15 mm polystyren, petri dish, USA Scientific Inc) were seeded within 2 ml of 0.4% top agar medium on top of the solidified base layer in the presence of ML385 (5 μM) or DMSO. The top layer was then covered with complete RPMI/10% FBS with 5 μM ML385 treatment or DMSO control. The plates were then incubated at 37°C in a 5% CO_2_ incubator for 20 days. Media were removed and colonies were fixed for 10 min at room temperature with 10% Methanol/10% glacial acetic acid. Colonies were stained with 2 ml of 0.014% crystal violet solution (Sigma‐Aldrich, St. Louis, Missouri, USA) for 2 h at room temperature, then washed with distilled water on a rocking shaker to clear the agar. Colonies were imaged using ZEN imaging software and counted using ImageJ.

### 2.8 In vitro cell viability assays

ML385 cytotoxicity was evaluated in MGH7 cells by seeding cells at (4 × 10^3^) per well in 96‐well plates. Cells were then treated with different concentrations of ML385 for 72 h. To determine the difference in cell viability between BKM120 treatment alone versus combined treatment with ML385 (5 μM) in MGH7 cells, 4 × 10^3^ cells were seeded in 96‐well plates for 24 h and then treated with a range of concentrations of BKM120 alone or in combination with ML385 (5 μM) for 72 h. Cell viability was quantified using the CellTiter 96® AQueous One Solution Reagent (Promega). Drug‐response curves were plotted and IC_50_ values were calculated using Graphpad Prism 6.0 for Windows (GraphPad Software, www.graphpad.com [RRID: SCR_002798]).

### 2.9 Clonogenic assays

Exponentially growing MGH7 cells were counted, diluted, and seeded in triplicate at 300 cells/well in 6‐well plates. Cells were incubated at 37°C for 24 h in a humidified CO_2_ incubator and then exposed to drugs or vehicle for 72 h. To assess clonogenic survival following drug exposure, cells were cultured in complete growth medium at 37°C for 11–14 days and then stained with 50% methanol‐crystal violet solution. Only colonies with more than 50 cells were counted.

### 2.10 In vivo growth of tumor xenografts

Male NOD/SCID mice were purchased from the MBB (MaxBell Basement) Animal Resource Centre of UHN. MGH7‐parent and MGH7‐NRF2 knockdown cells (2 × 10^6^ cells) were collected, resuspended in 150 μl PBS, mixed with 10% Matrigel at 4°C (Becton Dickinson Biosciences), and implanted into the subcutaneous flank of 4‐to 6 week‐old mice (6 mice per each arm) under anesthesia to create the two arms of the study. Starting at 8 days after implantation, tumor growth was monitored once or twice weekly by caliper measurements until the day that mice were sacrificed. Tumor volume was calculated with the following formula: volume (mm^3^) = length × width × height × 0.5.

### 2.11 Statistical analysis

Statistical significance between the control and the treatment groups was calculated either by using two‐sample Student's *t* test or two‐way ANOVA (GraphPad Prism 6, RRID: SCR_002798), as indicated in the figure legends. A *p*‐value of 0.05 or less was considered statistically significant.

## 3 RESULTS

### 3.1 Knockdown of NRF2 inhibits LUSC tumor formation in vivo

**FIGURE 1:**
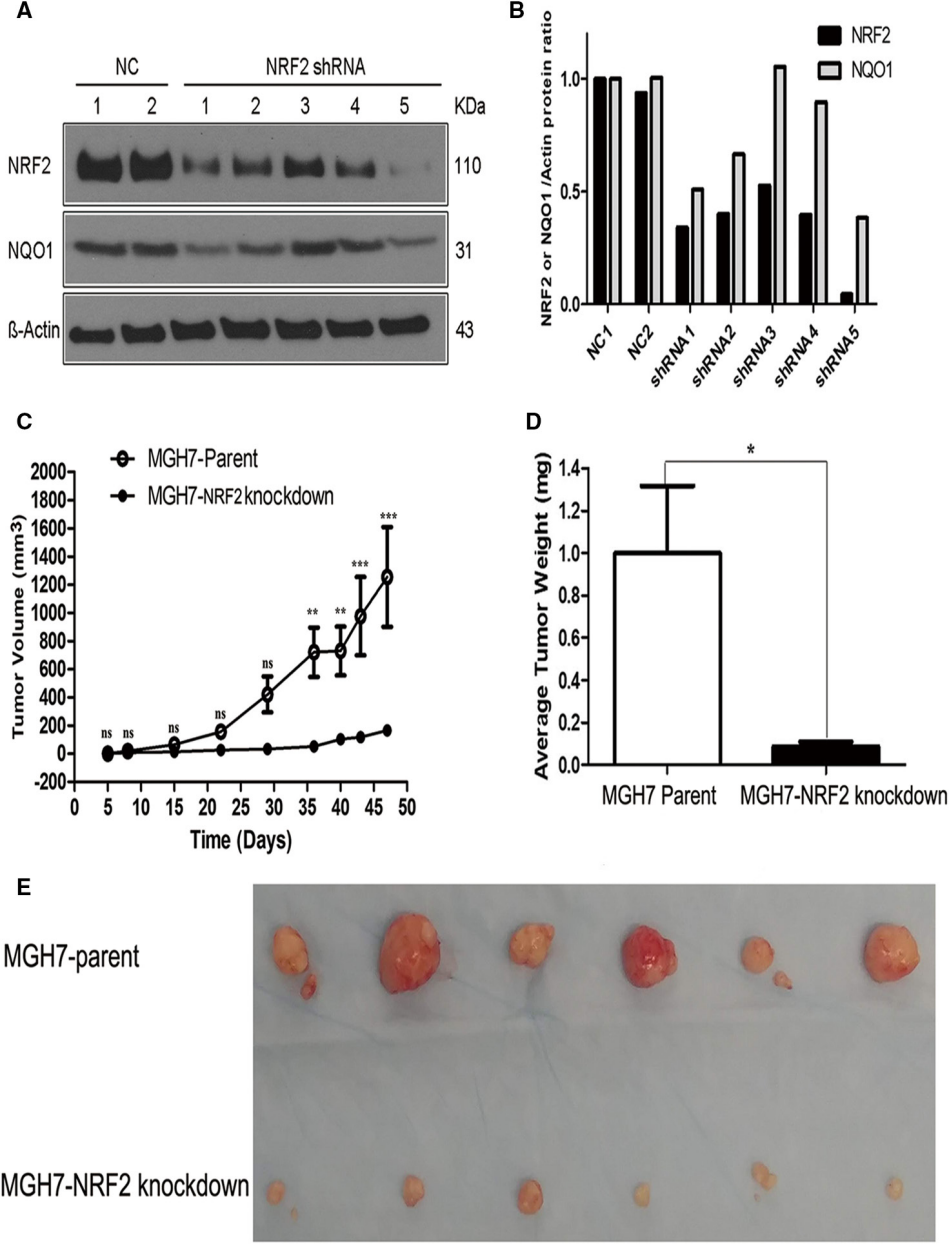
Knockdown of NRF2 inhibits proliferation of LUSC cells in vivo.

The dependency of human LUSC cancer cell growth on NRF2 under more physiologic conditions has not previously been investigated. To address this question, we first used the MGH7 LUSC cell line, which was established in our laboratory and expresses high levels of TP63 and SOX2, and recapitulates the squamous histology of the patient tumor when injected into immunocompromised mice.^31^, ^48^ MGH7 cells also express high levels of NRF2 protein, thus belonging to a class of cancer cells with apparent NRF2 activation.^49^ After screening five different NRF2 shRNA lentiviral constructs, we identified the shRNA5 construct as being especially effective at reducing protein levels of NRF2 and the NRF2 target gene, NQO1 in MGH7 cells (Figure 1A,B). Equivalent numbers of MGH7‐parental and MGH7‐NRF2 shRNA5 knockdown cells were injected into NOD/SCID mice. At the endpoint of the experiment, parental cells had generated tumors ~1200 mm^3^ in size, while tumors from NRF2‐knockdown cells had only grown to ~200 mm^3^ (Figure 1C). The volumes, weights, and sizes of the MGH7‐NRF2 knockdown tumors were all significantly smaller than in the parental group (Figure 1C–E), demonstrating decreased growth of LUSC cancer cells upon NRF2 inhibition in vivo.

### 3.2 The XDO377 LUSC organoid shows features of clinical LUSC with NRF2 activation

**FIGURE 2:**
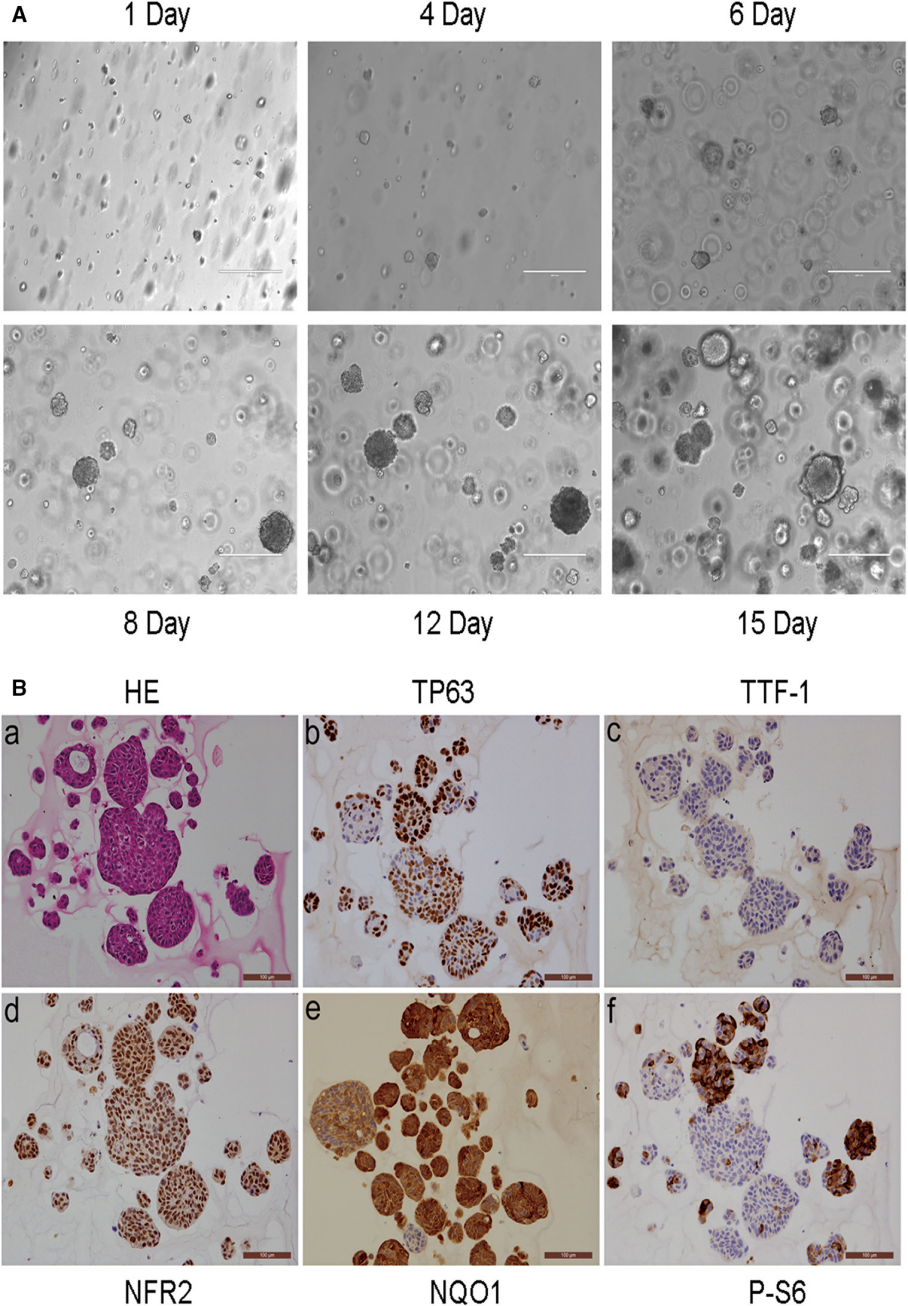
The XDO377 LUSC organoid shows features of clinical LUSC with NRF2 activation.

We have established NSCLC organoids, including the XDO377 LUSC model, from patient tumor and PDX models, which generally recapitulate the genomics and biology of patient tumors.^37^ As shown in Figure 2A, XDO377 organoids can be cultured without changes in their spherical morphology while maintaining their proliferation capacity. H&E and IHC analysis demonstrated that these LUSC organoids exhibit the morphological features common to squamous lung cancer tissues, including formation of intercellular bridges (Figure 2B a), and they stained positive for TP63 and negative for TTF‐1, which are immunophenotypes of LUSC (Figure 2B b,c).^50^ They also expressed high levels of NRF2 and its target gene NQO1 (Figure 2B d,e), which indicated that NRF2 signaling was active in these LUSC organoids. Ribosomal protein S6 phosphorylation was also positive (Figure 2B f). Overall, these data indicate that XDO377 retains key biological properties observed in LUSC and shows apparent NRF2 activation.

### 3.3 The small molecule NRF2 inhibitor, ML385, inhibits growth of LUSC organoids and cell lines in vitro

**FIGURE 3:**
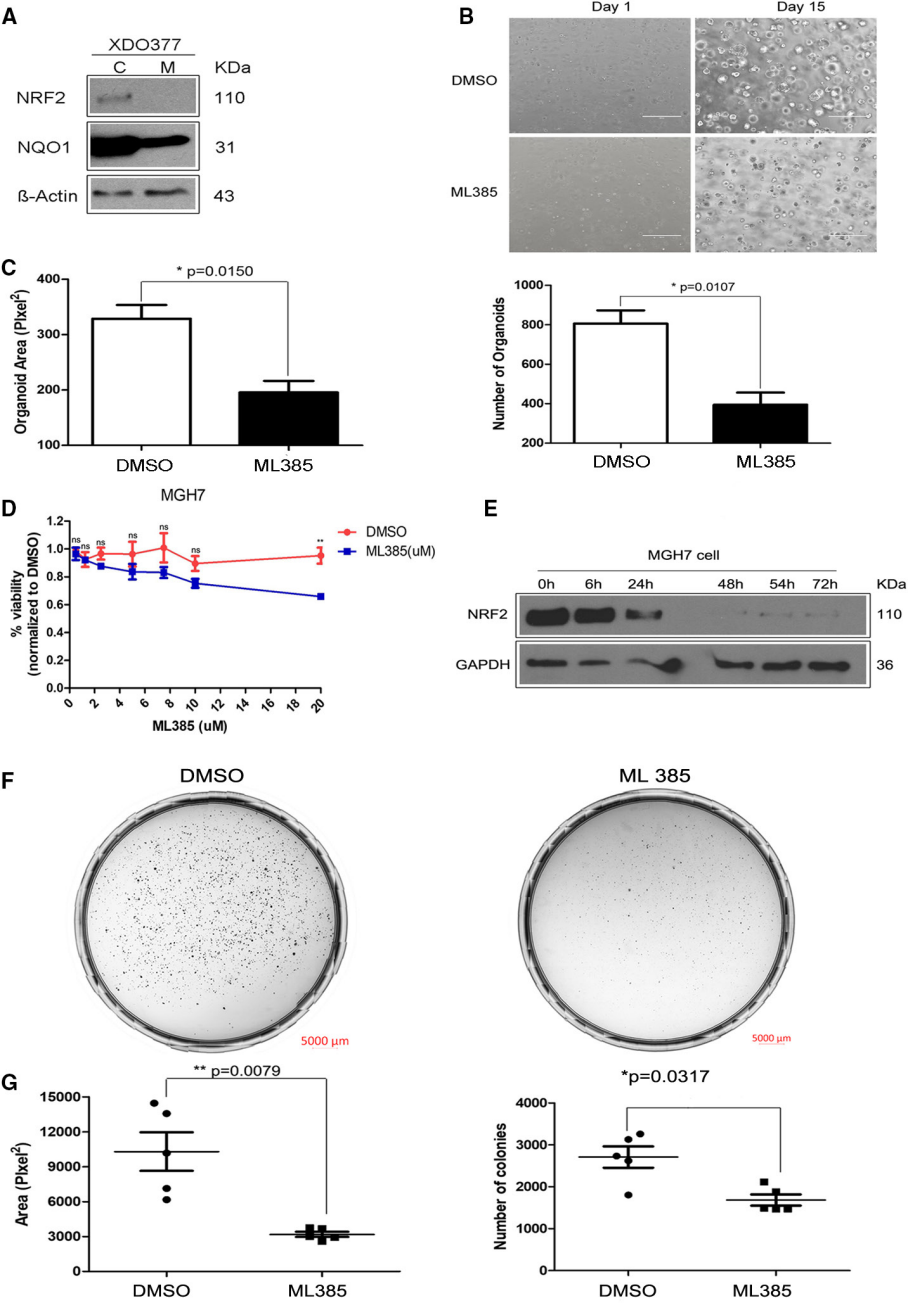
ML385 inhibits growth of LUSC organoids and LUSC cell lines in vitro.

We next investigated whether a small molecule NRF2 inhibitor can suppress growth of XDO377 LUSC organoids. For these experiments, we used ML385, which has been shown to preferentially inhibit NRF2 activity in cancer cell lines with NRF2 pathway activation.^38^ In agreement with previous data showing that NRF2 autoregulates its own transcription,^51^ we found that 5 μM ML385 treatment for 48 h inhibited expression of NRF2 and NQO1 in XDO377 cells (Figure 3A; Figure S1A). After 15 days of ML385 treatment, drug‐treated organoids were smaller and less numerous than the vehicle‐treated control cultures (Figure 3B,C). Collectively, these results support the in vivo findings with MGH7 cells and indicate that LUSC cells that express NRF2 are dependent on this transcription factor even when growing in 3D environments in vitro.

We also tested the in vitro sensitivity of MGH7 cells to ML385 when growing on standard 2D plastic. To exclude the possibility that potential sensitivity to ML385 treatment can be attributed to acute cytotoxicity, we examined MGH7 cell viability across different ML385 doses over 72 h. There was no significant drug toxicity of ML385 with concentrations as high as 5 μM or 10 μM (Figure 3D). This lack of cytotoxicity is also consistent with previous findings that growth of nontransformed immortalized lung epithelial cells is not inhibited by ML385 doses as high as 25 μM.^38^ 5 μM ML385 also inhibited NRF2 expression in MGH7 cells (Figure 3E and Figure S1B), as well as colony formation. Inhibition of colony formation was evident from the representative images shown in Figure 3F, and quantification of the number and sizes of the colonies (focusing on colonies >50 μm in size) (Figure 3G). Thus, in LUSC cells with NRF2 activation, NRF2 promotes growth under a variety of in vitro and in vivo conditions, and a small molecule NRF2 inhibitor can be used to interfere with their growth.

### 3.4 NRF2 promotes PI3K‐mTOR signaling in LUSC cells

**FIGURE 4:**
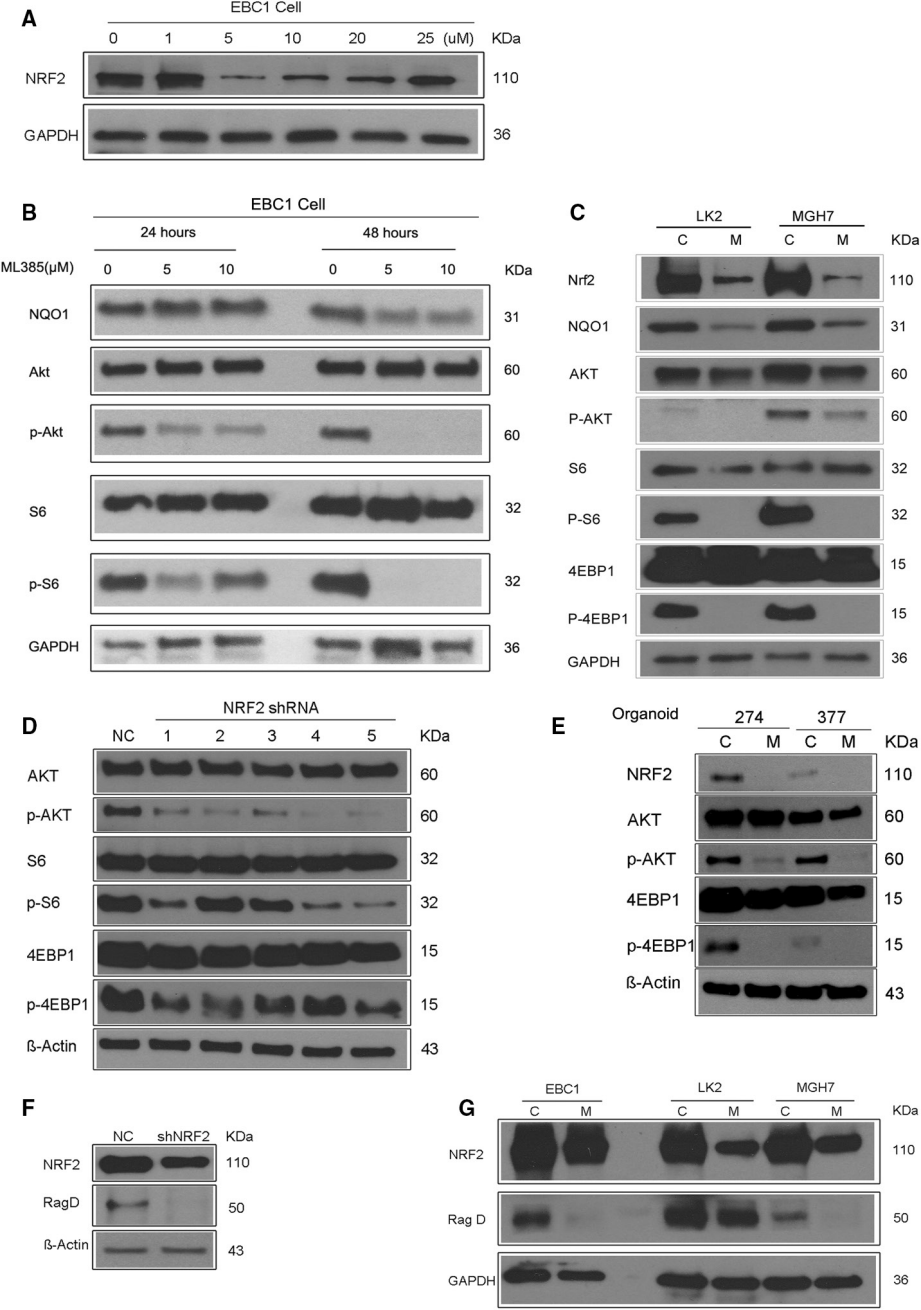
NRF2 promotes PI3K‐mTOR signaling in LUSC cells.

NRF2 promotes PI3K‐mTOR signaling in a variety of cancer cell line models.^7^, ^18^, ^19^, ^21^, ^22^, ^23^, ^24^ In these studies, NRF2 most consistently stimulated phosphorylation of the ribosomal protein S6, a target of the mTORC1 substrate, S6 kinase.^19^, ^22^, ^23^, ^24^ While ribosomal protein S6 phosphorylation was also shown to be regulated by NRF2 in the LUSC cell lines LK2 and EBC1, other points in the PI3K pathway were not investigated in these cells.^19^ Thus, we used several LUSC models, including some that are dependent on NRF2 for growth, to further characterize where NRF2 may activate the PI3K pathway in LUSC. We also wanted to determine if a small molecule NRF2 inhibitor can interfere with NRF2's ability to activate PI3K signaling. We first examined the effects of ML385 in the control LUSC cell lines, EBC1 and LK2, where NRF2 was initially connected to mTOR activation through upregulation of *RagD* mRNA levels.^19^ Both of these cell lines express NRF2 protein and harbor the stabilizing *NRF2* mutations, E79K and D77V, respectively.^11^ Consistent with the results in MGH7 cells, we found that in EBC1 cells, treatment with 5 μM ML385 for 48 hours inhibited NRF2 expression (Figure 4A and Figure S2A). Unexpectedly, we noticed that in EBC1 cells, as the ML385 concentration rose beyond 5 μM, NRF2 protein levels started to recover. This could reflect the increase in intracellular ROS that presumably occurs due to the initial reduction in NRF2 levels by ML385, which could then act post‐transcriptionally to increases NRF2 protein stability.^52^ Treatment of EBC1 cells with 5 μM ML385 also reduced NQO1 expression, as well as phosphorylation of AKT and the ribosomal protein S6, especially at 48 h post‐treatment (Figure 4B and Figure S2B). Similar results were also observed after ML385 treatment (5 μM, 48 h) of LK2 and MGH7 cels (Figure 4C, and Figure S2C). We additionally determined that ML385 reduced 4EBP1 phosphorylation in these two LUSC cell lines, a direct target of mTORC1, further supporting that PI3K and mTOR activity are diminished by drug treatment (Figure 4C, and Figure S2C). We then used multiple distinct NRF2 shRNA lentiviruses in MGH7 cells to address whether the effect of ML385 on PI3K‐mTOR signaling was due to an “on‐target” effect. Relative to a control shlacz virus, multiple NRF2 shRNA viruses reduced phosphorylation of AKT, ribosomal protein S6, and 4EBP1 (Figure 4D, and Figure S2D). Finally, we found that ML385 also inhibited AKT and 4EBP1 phosphorylation in two LUSC organoid models, XDO274 and XDO377 growing in 3D (Figure 4E, and Figure S2E). Overall, although NRF2 promotes mTORC1 activity, it also acts upstream of mTORC1 at the level of AKT or higher in LUSC cancer cells.

It has previously been shown in LUSC cell lines that NRF2 positively regulates mRNA levels of the mTORC1 activator, *RagD*, and that in patient LUSC samples, *NRF2* activating mutations are correlated with increased *RagD* mRNA expression.^19^, ^21^ Since we found evidence for NRF2 acting upstream of mTORC1, it is possible that in our models, NRF2 either acts exclusively upstream of mTORC1 such as at the level of PI3K or AKT, or both upstream and at the level of mTORC1, possibly through RagD. To distinguish between these possibilities, we first tested whether knockdown of NRF2 by shRNA in MGH7 cells affected RagD expression. NRF2 knockdown reduced RagD protein expression (Figure 4F, and Figure S2F), indicating that the *RagD* mRNA regulation previously reported in the EBC1 and LK2 LUSC cell lines^19^ extends to RagD protein levels in other LUSC models. This regulation likely reflects an on‐target effect of the NRF2 shRNA since RagD levels were also reduced in MGH7 cells and the positive control cell lines EBC1 and LK2 by ML385 (Figure 4G, and Figure S2G). Thus, NRF2 may affect PI3K‐mTOR signaling at two different points, with one point being at the level of mTORC1 through RagD, as previously reported.^19^

Several studies, including one with a LUSC cell line model, found that PI3K‐AKT signaling can also cross‐talk with NRF2 to promote its stabilization.^16^, ^53^, ^54^, ^55^, ^56^, ^57^, ^58^ However, we could not confirm this as a general finding in LUSC. In EBC1 cells, the PI3K inhibitor BKM120 reduced phosphorylation of AKT and ribosomal protein S6, but did not affect NRF2 protein levels (Figure S3).

### 3.5 ML385‐mediated downregulation of RagD is correlated with reduced recruitment of mTORC1 to the lysosome

**FIGURE 5:**
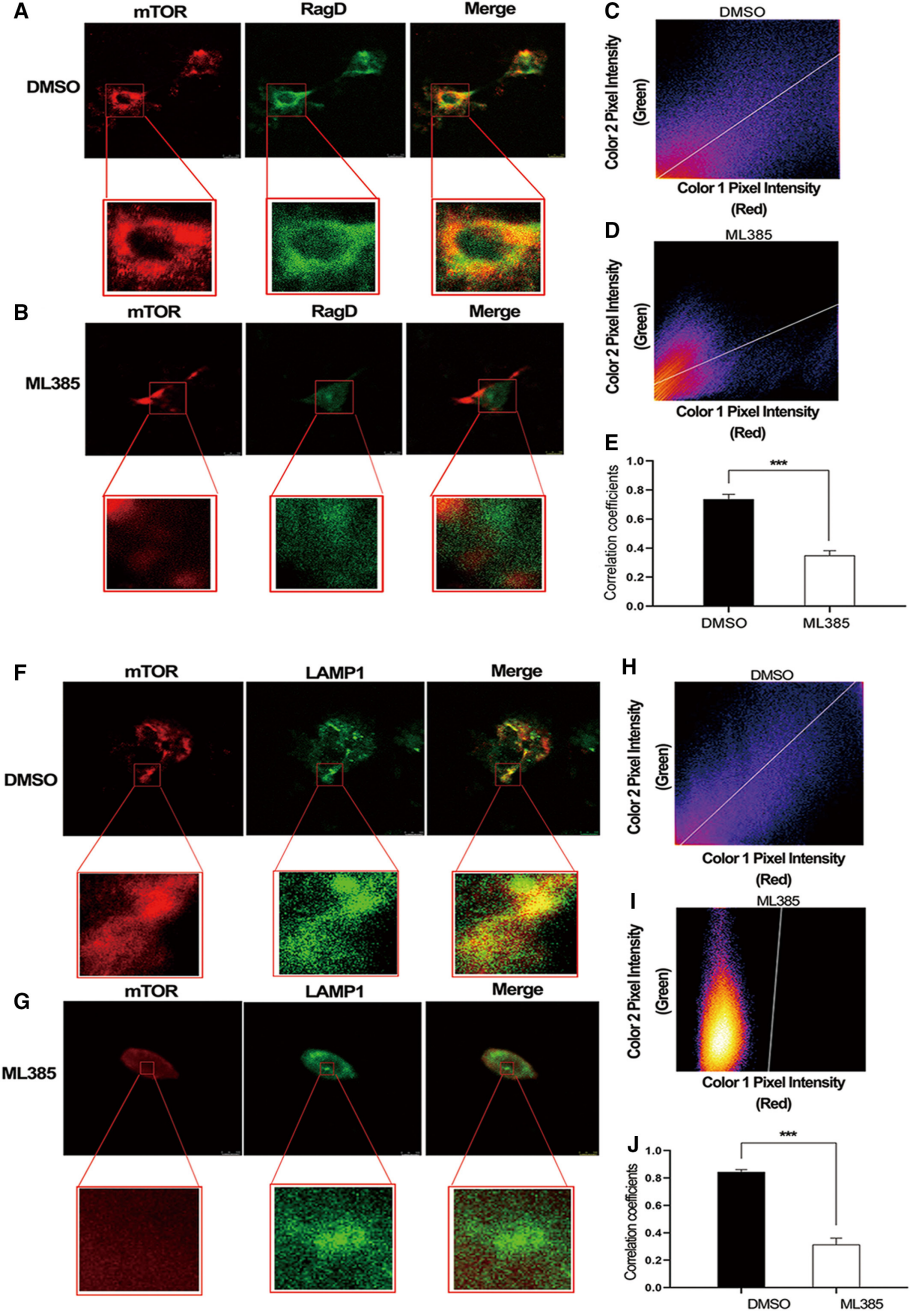
ML385 inhibits recruitment of mTOR to the lysosome.

Rag GTPases, including RagD, are essential for nutrient‐induced recruitment and activation of mTORC1 at lysosomes.^29^, ^59^, ^60^ Although NRF2 was previously shown to increase phosphorylation of proteins downstream of mTORC1 while also increasing *RagD* mRNA levels, it was unclear whether this regulation of RagD by NRF2 affects mTOR recruitment to lysosomes.^19^ To address this question, we used ML385 to inhibit NRF2 activity in LK2 LUSC cells, and then starved and stimulated the cells with amino acids to examine the effect of ML385 on mTOR localization. ML385 treatment decreased the fluorescence intensity of RagD (Figure 5B compared with Figure 5A, Figure S4B compared with Figure S4A), consistent with the reduction in its protein levels seen by Western blotting (Figure 4G, and Figure S2G). In addition, we used the coloc 2 plugin in Fiji software to visualize the corresponding scatter plots for the fluorescence signals and determine the PCC of the different colocalization situations. For example, the scatter plot shown in Figure 5C corresponds to the zoomed in area of the control cell shown in Figure 5A, and revealed a PCC of 0.59 for the mTOR and RagD signals. By contrast, the scatter plot in Figure 5D for the zoomed in area of the ML385‐treated cell shown in Figure 5B showed a reduced PCC of 0.39. We then quantified the PCC data from 10 cells per condition (Figure S4A, and Figure S4B). As shown in Figure 5E, the mean PCC value for mTOR and RagD colocalization decreased from 0.738 to 0.35 (*p* < 0.001). Using LAMP1 as a lysosomal marker, we also found that after amino acid stimulation, ML385 reduced mTOR recruitment to lysosomes (Figure 5F,G). Similar to the disruption of mTOR and RagD colocalization, coloc 2 analysis revealed that ML385 also reduced mTOR and lysosome colocalization for the cells shown in Figure 5F,G, with the PCC being reduced from 0.74 (Figure 5H) to 0.09 (Figure 5I). Across 10 cells per condition, ML385 caused the mean PCC for mTOR and lysosomal colocalization to decrease from 0.845 to 0.315 (Figure S4C,D and Figure 5J, *p* < 0.001). Thus, the changes in RagD expression caused by ML385 appear to impact mTOR recruitment and activation at lysosomes.

### 3.6 ML385 cooperates with BKM120 to more strongly suppress PI3K signaling and growth of LUSC cells

**FIGURE 6:**
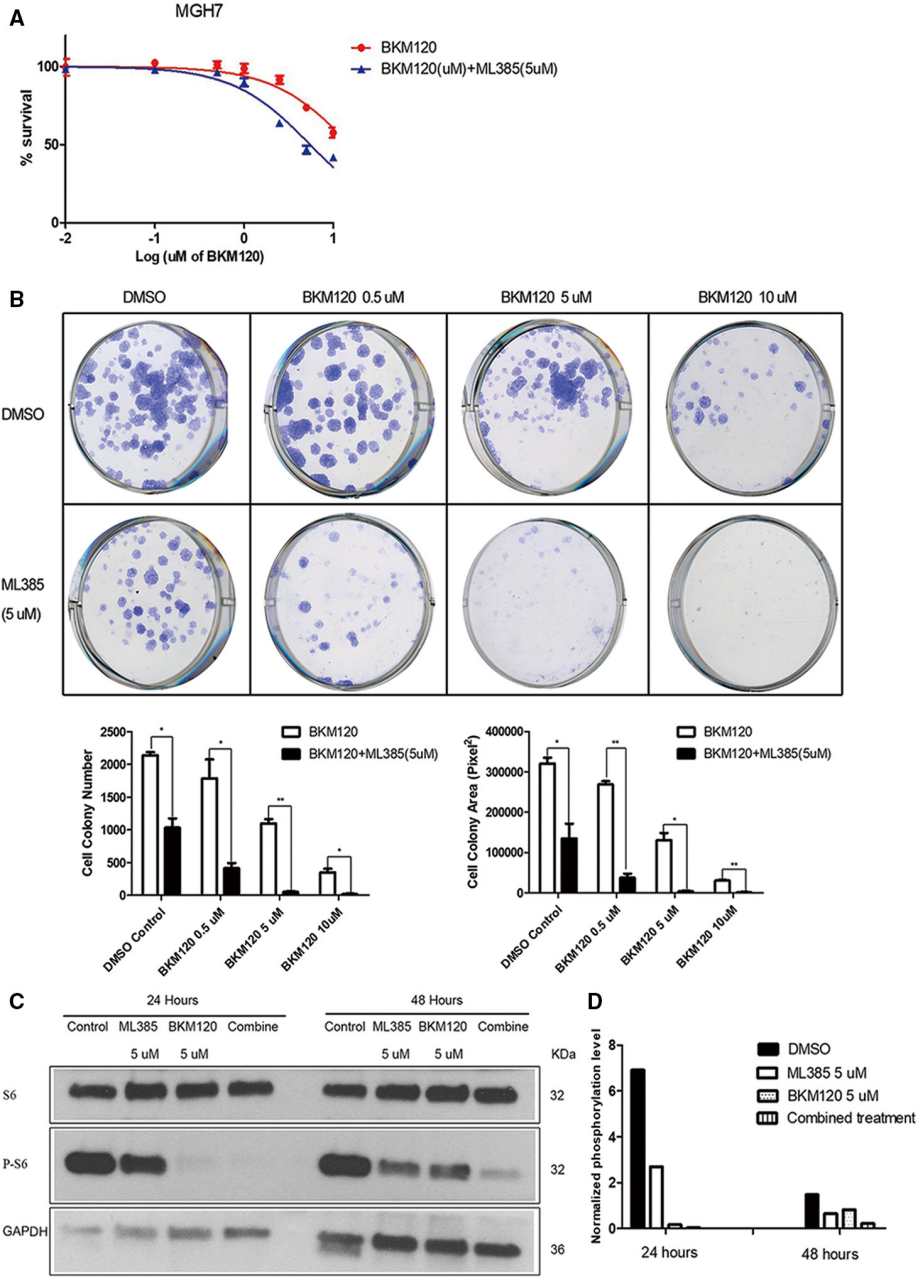
ML385 cooperates with BKM120 to more strongly suppress PI3K signaling and growth of MGH7 cells.

PI3K inhibitors such as BKM120, which targets all four catalytic isoforms of class I PI3K (p110α, p110β, p110δ and p110γ),^61^ have been evaluated in the clinic for LUSC patients.^62^ BKM120 monotherapy was not successful in LUSC patients preselected for genetic alterations in the PI3K pathway.^62^ However, given that NRF2 also regulates PI3K signaling, we explored whether a NRF2 inhibitor could sensitize LUSC cancer cells to PI3K inhibitors such as BKM120. Treatment of MGH7 cells with 5 μM ML385 reduced the IC50 of BKM120 from 15.46 μM to 5.503 μM (Figure 6A) and in a clonogenic assay, enhanced the ability of BKM120 to inhibit colony formation (Figure 6B). Western blotting indicated that combination of BKM120 with ML385 reduced the partial rebounding of mTORC1 signaling that was seen at 48 h after treatment with BKM120 alone (Figure 6C,D). Thus, small molecule NRF2 inhibitors may improve sensitivity to PI3K inhibitors in LUSC patients.

## 4 DISCUSSION

NRF2 is a master regulator of antioxidant responses and its activation is thought to be key to the survival of multiple types of cancer.^63^ It is most frequently activated genetically in LUSC (25% altered), suggesting an especially prominent role in this disease.^25^ Earlier work found that NRF2 promotes growth of LUSC cell lines in vitro. However, it was unknown whether this is also true in vivo and in LUSC models that more closely mimic patient disease when growing in vitro. Here, we report that knockdown of NRF2 in the MGH7 LUSC cell line inhibits tumor formation in vivo. We further show that the small molecule NRF2 inhibitor ML385 inhibits proliferation of an LUSC organoid in vitro while growing in a complex 3D environment. These data support the notion that NRF2 promotes the growth of LUSC in vivo and is not just required for growth in cell lines that have been adapted to 2D cell culture.

NRF2 can affect cancer cell growth through multiple mechanisms. One mechanism by which NRF2 controls cell proliferation is through the regulation of reactive oxygen species (ROS). NRF2 is recognized as the master regulator of cellular antioxidant responses through its ability to restore redox homeostasis. Thus, cancer cells are able to thrive despite having high ROS levels by constitutively activating NRF2.^64^ NRF2 can also affect cancer cell cycling by promoting M‐phase entry.^65^, ^66^ Additionally, cancer cells have higher energetic and anabolic needs to support their rapid cell growth, and NRF2 contributes to the fulfillment of these demands. In this regard, NRF2 can partner with ATF4 to promote activation of the serine/glycine biosynthetic pathway.^67^ Also, NRF2 can redirect glucose and glutamine into anabolic pathways, an activity which is augmented by sustained activation of the PI3K‐AKT pathway.^7^ The PI3K pathway is also commonly activated through genetic alterations in LUSC, including by point mutations in *PIK3CA* ^14^. The PI3K pathway increases NRF2 protein levels through several mechanisms, most notably, by inhibiting its degradation through both the KEAP1 and SCF/β‐TrCP pathways. This is accomplished through AKT‐mediated phosphorylation and inhibition of GSK3β, which drives the SCF/β‐TrCP pathway,^53^, ^56^ as well as AKT and mTORC1‐mediated phosphorylation of p21 and p62, respectively, which interfere with KEAP1‐dependent NRF2 degradation.^55^, ^57^, ^58^ Surprisingly, however, in EBC1 LUSC cells, we did not detect regulation of NRF2 levels by PI3K signaling, which contrasts with what was reported in the LC‐1/SQSF LUSC cell line.^68^ Therefore, there may be some nuances to when and how PI3K signaling affects NRF2 expression.

Another mechanism through which NRF2 promotes cancer cell growth is through upregulation of PI3K‐mTOR signaling.^7^, ^18^, ^19^, ^21^, ^22^, ^23^, ^24^ We also find that NRF2 regulates PI3K‐mTOR signaling in multiple LUSC models, including organoids growing in 3D, as evidenced by changes in phosphorylation of AKT, ribosomal protein S6, and 4EBP1 after treatment with either NRF2 shRNA or ML385. Our data agree with prior findings that all consistently found NRF2 promotes ribosomal protein S6 phosphorylation, including in several of the LUSC cell lines used in our study, LK2 and EBC1.^19^, ^22^, ^23^, ^24^ Fewer studies have examined AKT phosphorylation, but studies in cultured melanocytes and in mouse liver also reported NRF2 stimulates AKT phosphorylation,^7^, ^22^ while overexpression of NRF2 in HEK293 cells did not affect AKT.^19^ Thus, collectively, our data and prior work largely support NRF2 generally stimulating AKT and mTORC1 activity across a variety of normal and cancer cell types.

If NRF2 acted exclusively at the level of or upstream of AKT, it would be expected that activity of the mTORC1 complex would also be affected. However, while we could not determine the mechanism through which NRF2 stimulates AKT phosphorylation, we did confirm that NRF2 may at least additionally act downstream of AKT, through RagD. The Rag GTPasess are obligate heterodimers of RagA or RagB with RagC or RagD, which are essential to recruit and activate the mTORC1 complex at the lysosomal membrane after amino acid stimulation.^29^, ^59^ NRF2 was previously shown to upregulate *RagD* mRNA levels in the LUSC cell lines EBC1 and LK2,^19^ and *NRF2* activating mutations are significantly associated with increased *RagD* mRNA expression in LUSC patient tumors.^21^ We now confirm this finding at the protein level in these cell lines, as well as MGH7 LUSC cells. We further show that this increase in protein expression in LK2 cells is likely to be functionally important for promoting mTOR recruitment to lysosomes, where mTORC1 can be activated following amino acid stimulation. These data support the notion that although NRF2 may act at or upstream of AKT, the upregulation of RagD by NRF2 likely also contributes to increased mTORC1 signaling in LUSC. Indeed, consistent with this idea, upregulation of only RagD by MiT/TFE transcription factors has been proposed to be sufficient to increase mTORC1 activity in a variety of settings.^60^

LUSC accounts for approximately 400,000 deaths per year worldwide.^10^ However, no agents that specifically target driver mechanisms have yet been approved for its treatment. Our work supports continued attempts to target NRF2 in LUSC, especially because of the potential of NRF2 inhibitors to simultaneously antagonize multiple pro‐oncogenic activities including PI3K‐mTOR signaling, antioxidant responses, and metabolism. In particular, development of more potent ML385 derivates with good pharmacokinetics and bioavailability should be pursued.

## AUTHOR CONTRIBUTIONS

**Lili Ji:** Conceptualization (lead); data curation (equal); formal analysis (equal); funding acquisition (equal); methodology (equal); resources (supporting); writing – original draft (lead); writing – review and editing (lead). **Nadeem Moghal:** Conceptualization (supporting); funding acquisition (equal); resources (supporting); writing – original draft (equal); writing – review and editing (equal). **Xinru Zou:** Data curation (equal); formal analysis (equal); writing – review and editing (supporting). **Yixuan Fang:** Data curation (equal); formal analysis (equal); writing – review and editing (supporting). **Shuning Hu:** Data curation (equal); formal analysis (equal); writing – review and editing (supporting). **Yuhui Wang:** Data curation (equal); formal analysis (equal); writing – review and editing (supporting). **Ming‐Sound Tsao:** Conceptualization (equal); funding acquisition (lead); project administration (lead); resources (lead); supervision (lead); writing – review and editing (equal).

## FUNDING INFORMATION

The study was partially supported by the Canadian Institutes of Health Research (CIHR) (grant FDN‐148395 to Ming‐Sound Tsao), Canadian Institutes of Health Research (CIHR) (grant PJT‐175190 to Nadeem Moghal), Nantong Science and Technology Project (JC2019138 to Lili Ji), and Natural Science Program of Nantong University School of Medicine (TDYX2021003 to Lili Ji).

## CONFLICT OF INTEREST

All authors declare that there are no conflicts of interests.

## ETHICAL APPROVAL STATEMENT

The use of resected NSCLC specimens for this study and for the establishment of PDXs and organoids was approved by the University Health Network (UHN) Research Ethics Broad (REB 09–0510 and 17–5518) and was previously reported.^37^, ^40^ Experiments using the PDX models followed a protocol approved by the UHN Animal Care Committee (AUP: 5555). The source of the cells used to develop the organoids were from surgically resected primary tumors from patients with early‐stage NSCLC. Informed written consent was received from all patients. All studies were performed in accordance with Canadian TRI‐Council Policy Statement: Ethical Conduct for Research Involving Humans. All animal studies were performed under an animal use protocol (AUP 4151) approved by the Animal Care Committee of the University Health Network, Toronto, Canada.

## Supporting information


Figure S1

Figure S2

Figure S3

Figure S4

## Data Availability

Data that supports the findings of this study are available from the corresponding author upon request.
