# Supplementary figures and images for: The NRF2 antagonist ML385 inhibits PI3K‐mTOR signaling and growth of lung squamous cell carcinoma cells
Source: Cancer Med. 2022 Oct 28;12(5):5688–702. doi: 10.1002/cam4.5311 (PMC10028163; doi:10.1002/cam4.5311)

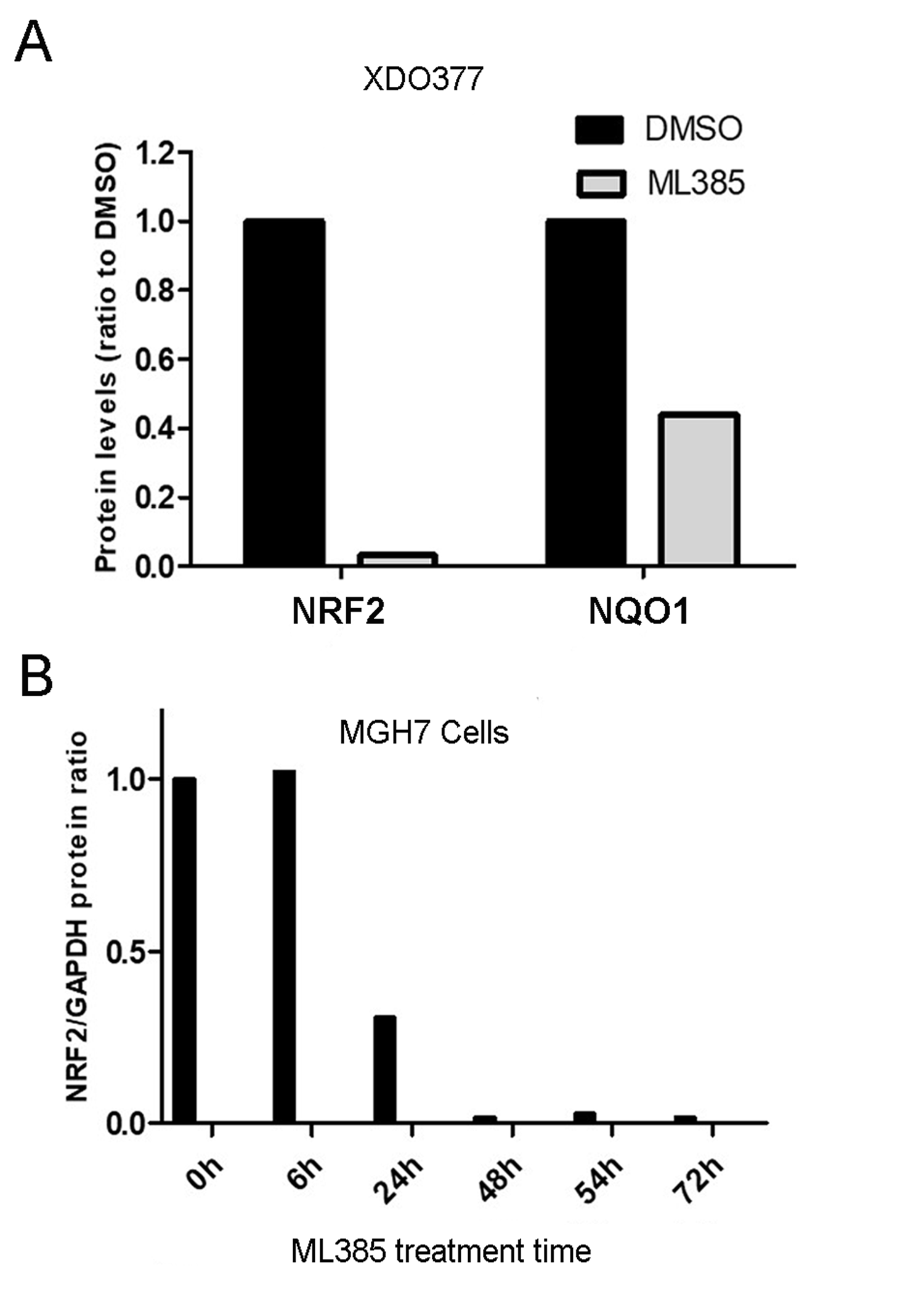

Supplement: Supplementary file 1 — Figure S1 [file CAM4-12-5688-s002.tif]

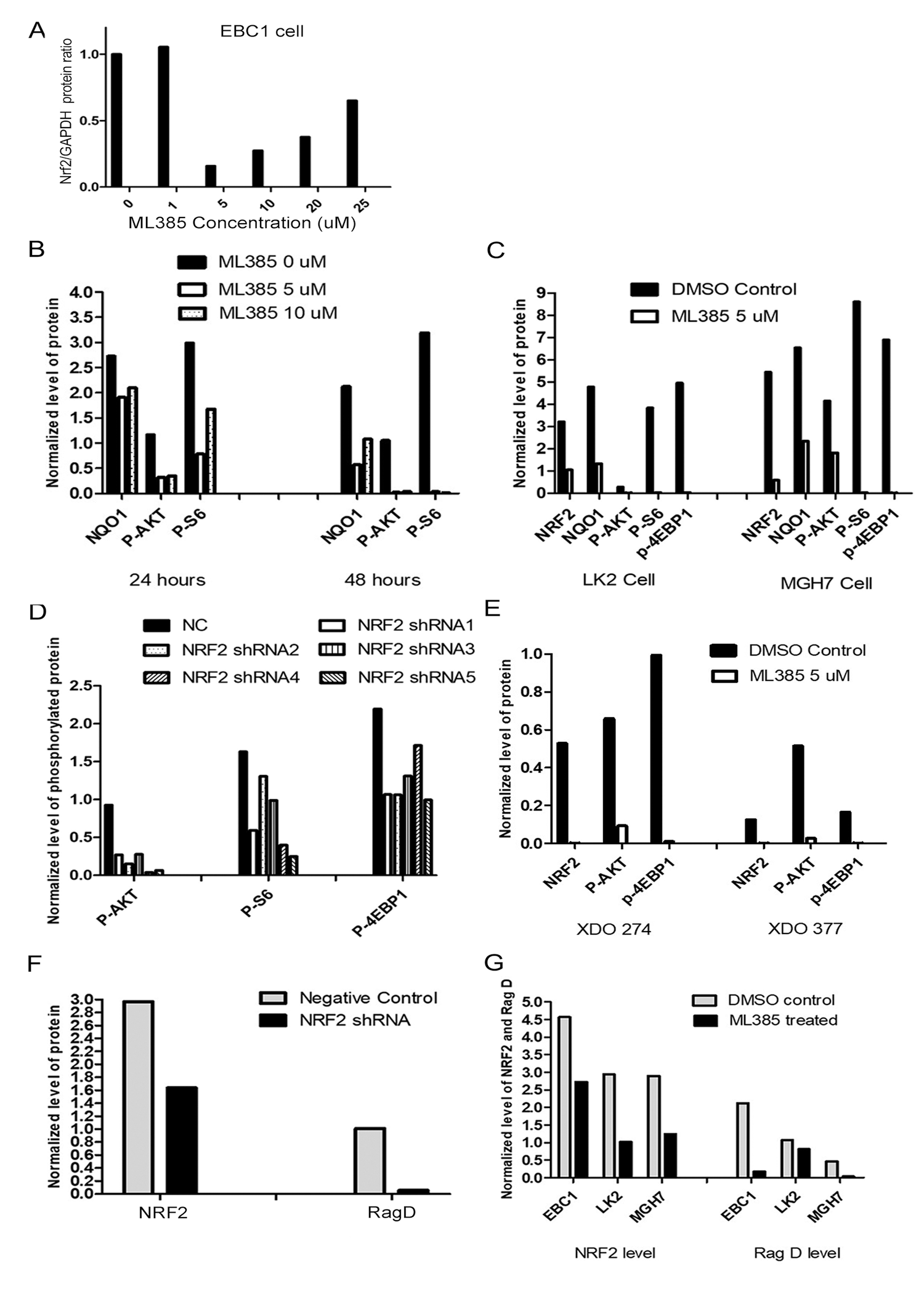

Supplement: Supplementary file 2 — Figure S2 [file CAM4-12-5688-s004.tif]

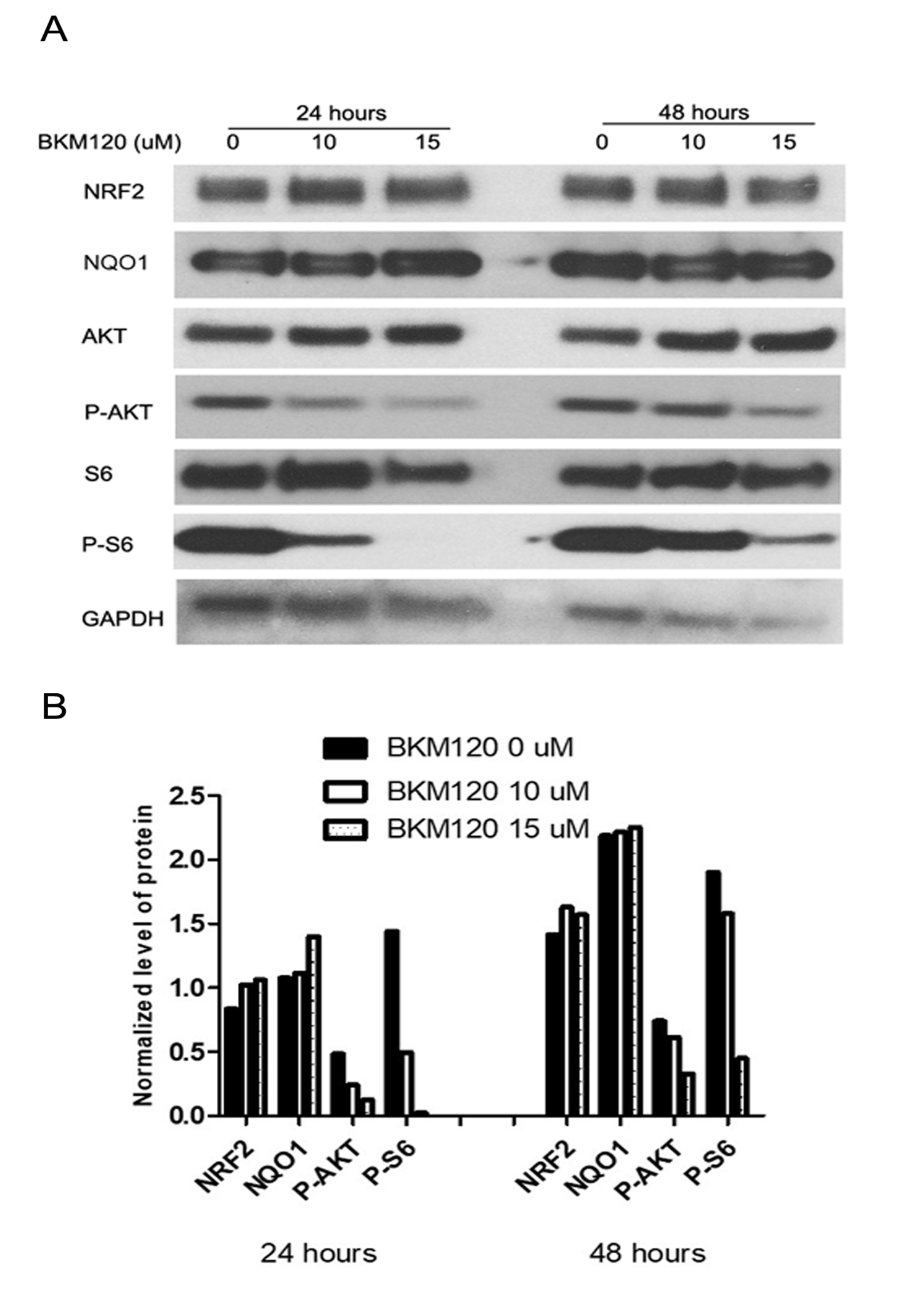

Supplement: Supplementary file 3 — Figure S3 [file CAM4-12-5688-s003.tif]

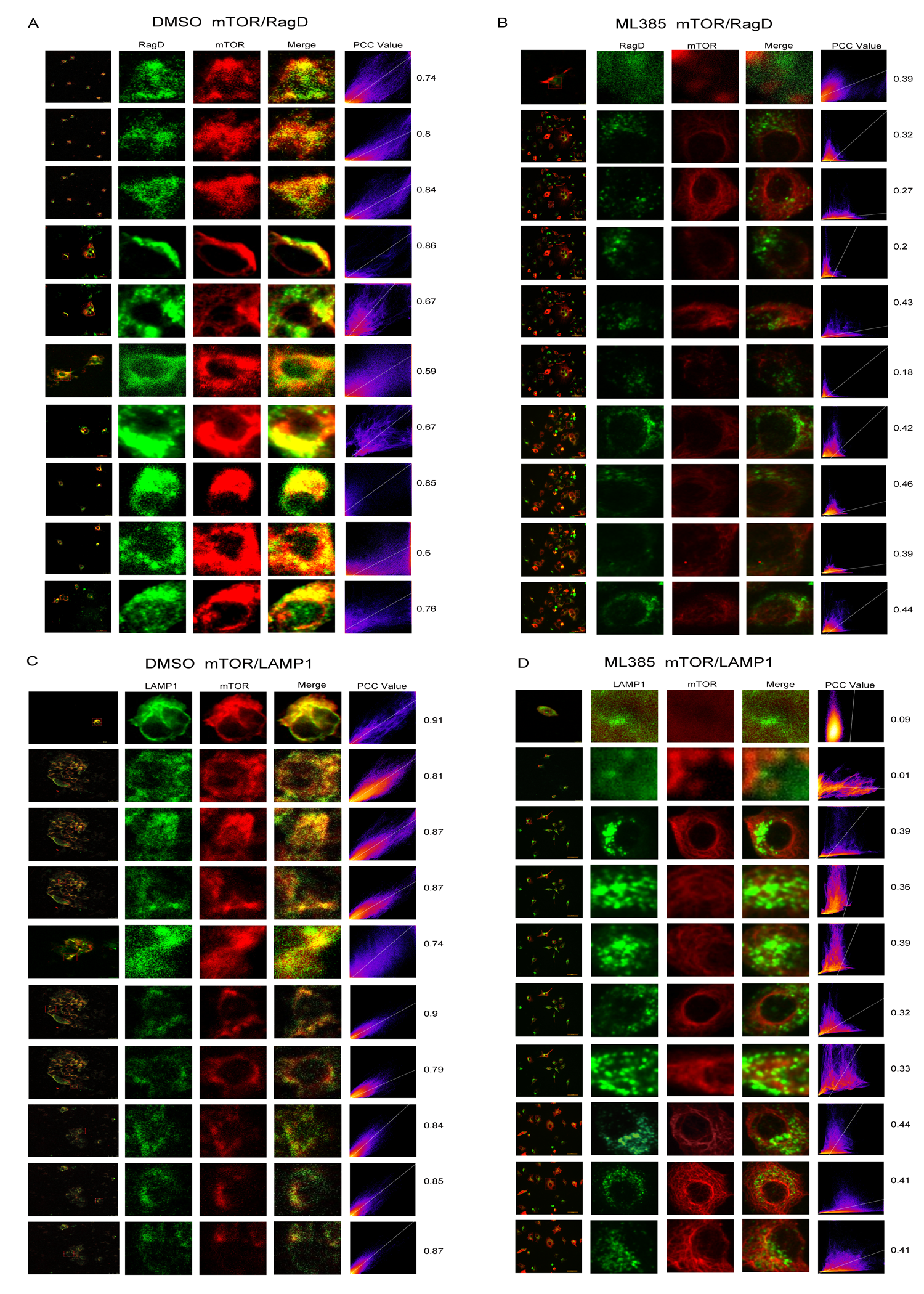

Supplement: Supplementary file 4 — Figure S4 [file CAM4-12-5688-s001.tif]
